# Long-term outcomes and prognostic factor analysis of resected Siewert type II adenocarcinoma of esophagogastric junction in China: a seven-year study

**DOI:** 10.1186/s12893-020-00926-1

**Published:** 2020-11-30

**Authors:** Yiding Feng, Youhua Jiang, Qiang Zhao, Jinshi Liu, Hangyu Zhang, Qixun Chen

**Affiliations:** 1 Department of Thoracic Surgery, Cancer Hospital of the University of Chinese Academy of Sciences(Zhejiang Cancer Hospital), Hangzhou, 31000 China; 2 Department of Cancer Biotherapy Center, First Affiliated Hospital, School of Medicine, Zhejiang University, Hangzhou, 31000 China

**Keywords:** Esophagogastric junction adenocarcinoma, Lymph nodes, Siewert classification, Surgery

## Abstract

**Background:**

The incidence rate of adenocarcinoma of the esophagogastric junction (AEG) has significantly increased over the past two decades. Surgery remains the only curative treatment. However, there are currently few studies on Chinese AEG patients. The purpose of this study was to retrospectively analyze the survival and prognostic factors of AEG patients in our center.

**Methods:**

Between January 2008 and September 2014, 249 AEG patients who underwent radical resection were enrolled in this retrospective study, including 196 males and 53 females, with a median age of 64 (range 31–82). Prognostic factors were assessed with the log-rank test and Cox univariate and multivariate analyses.

**Results:**

The 5-year survival rate of all patients was 49%. The median survival time of all enrolled patients was 70.1 months. Pathological type, intraoperative blood transfusion, tumor size, adjuvant chemotherapy, duration of hospital stay, serum CA199, CA125, CA242 and CEA, pTNM stage, lymphovascular or perineural invasion, and the ratio of positive to negative lymph nodes (PNLNR) were significantly associated with overall survival when analyzed in univariate analysis.

**Conclusions:**

Our study found that adjuvant chemotherapy, PNLNR, intraoperative blood transfusion, tumor size, perineural invasion, serum CEA, and duration of hospital stay after surgery had significance in multivariate analysis and were independent risk factors for survival.

## Background

Adenocarcinoma of the esophagogastric junction (AEG) has been reported to account for approximately 5–8% [1, 2] of all esophageal cancers in China and 35.7% of gastric cancers and lower esophageal cancers worldwide [3]. Many population-based studies have shown that the incidence rate of AEG has significantly increased over the past two decades, both in Western countries and in East Asia [4–6]. The reported seven-fold increase in the morbidity rate of AEG [7], which is a more substantial increase compared to that other malignancies, has led to a steady increase in the mortality rate from 2–15/100,000 patients [8].

Surgical resection is the main curative treatment for AEG. Unlike the treatment for gastric cancer, which is standard surgical resection plus D2 lymph node resection, surgery for AEG is still controversial in many ways, especially for Siewert type II AEG. The surgical treatment of AEG includes primary tumor removal, lymph node dissection and reconstruction of the digestive tract. Regional lymph node metastasis is the most common metastasis method of AEG. Studies have found that the lymph node metastasis rate of AEG is 76.3%, much higher than that of distal gastric adenocarcinoma (67.4%) [9]. Moreover, the distribution of lymph node metastasis in different Siewert subtypes was different, resulting in several different surgical approaches [10, 11], and there are still controversies regarding the N staging of the AJCC TNM staging system [12]. Other controversial issues include the choice between laparoscopic surgery or open surgery and whether patients with AEG who achieve R0 resection should undergo neoadjuvant chemoradiotherapy.

Based on the above problems, although Siewert classified AEG in 2000, there are still many problems that exist in clinical practice. In China, squamous cell carcinoma remains the predominant pathological type of esophageal cancer. Therefore, esophageal adenocarcinoma has rarely been investigated among Chinese patients. Our study aimed to explore the long-term outcomes of AEG Chinese patients who underwent resection and to analyze the related prognostic factors.

## Methods

### Patients

From January 2008 to September 2014, there were 420 cases in which curative R0 resection was performed for esophagogastric junction cancers. Data from these 420 patients were collected, and the inclusion criteria were as follows: (I) patients with pathologically and immunohistochemically diagnosed AEG; (II) patients who underwent radical resection and did not have distant metastasis; and (III) patients with Siewert type II AEG. The exclusion criterion was patients without complete data for analysis. Among all collected patients, 249 patients had complete clinical data and confirmed postoperative pathology for adenocarcinoma and met all the inclusion and exclusion criteria.

Data on the demographics, comorbidities, pathologic details, surgical approach, blood infusion, duration of hospital stay, adjuvant therapy and survival time were collected and subsequently analyzed. Among the characteristics, the duration of hospital stay was classified according to whether the patient was hospitalized for longer than 10 days. The tumor size was classified according to whether the tumor size was larger than 4 cm; the tumor pathology was subdivided according to whether there was lymphovascular invasion or perineural invasion. Because the number of lymph nodes dissected varied greatly among different patients, we specifically defined the ratio of positive to negative lymph nodes for all dissected lymph nodes to further differentiate the prognostic ability of different values, defined as the PNLNR (positive lymph nodes/negative lymph nodes ratio).

All postoperative patients in our center were followed up regularly. In general, the frequency of follow-up was every 3 months for the first 2 years, then every 6 months for the following 3 years, and annually thereafter. Telephone follow-up interviews were conducted at irregular intervals. This retrospective study was performed in accordance with the ethical standards of the Ethics Committee of Zhejiang Cancer Hospital and received Institutional Review Board approval. No informed consent was needed for this study.

### Surgical approach selection

A reasonable surgical route should consider tumor resection, lymph node dissection, surgical margin and safety. The alternative AEG surgical approaches include left thoracotomy and the Ivor-Lewis, McKeown, and transhiatal or abdominal-transhiatal approaches. The selection of surgical approach referred to the NCCN guidelines for esophageal and esophagogastric junction cancers, NCCN guidelines for gastric cancer and Chinese expert consensus on the surgical treatment for adenocarcinoma of the esophagogastric junction. Preoperative staging was performed according to the eighth edition of the TNM staging system, and preoperative classification was performed based on the Siewert type.

In general, the abdominal-transhiatal approach was preferred for those with < 3 cm of esophagus involved, and the thoracotomy approach was preferred for those with ≥ 3 cm of esophagus involved.

### Statistical analyses

All statistical calculations were performed with IBM SPSS Statistics (Version 19.0; IBM Corp., New York, USA). Charts were made with GraphPad Prism 6 (GraphPad Software, Inc., La Jolla, CA, USA). Overall survival (OS) was calculated from the date of surgery to the date of death due to any cause. The data of patients lost to follow-up were censored at the date of the last observation. The Cox proportional hazards model was used to determine the univariate and multivariate hazards ratios for the study parameters. Pearson product-moment correlation analysis was used to measure the relationship between two variables. For all tests, P < 0.05 was defined as statistically significant.

## Results

### Characteristics of the patients

The baseline characteristics of the patients are shown in Tables 1, 2 and 3. There were 196 (78.7%) males and 53 (21.3%) females in the population. The median age of all enrolled patients was 64 years, ranging from 31–82 years. The details of the pathological outcomes showed that most patients (72.3%) had pure adenocarcinoma, and the remaining patients had mixed adenocarcinoma with signet ring or mucinous tumors. There were 165 (66.3%) patients with tumor diameters larger than 4 cm and 84 (33.7%) patients with tumor diameters less than or equal to 4 cm. Among them, 127 (51.0%) patients had lymphovascular invasion, and 154 (61.8%) patients had perineural invasion. Pathological differentiation showed that only 2 patients were classified as G1, 92 (36.9%) were classified as G2, and 155 (62.2%) were classified as G3.

**Table 1:** Clinical characteristics of 249 patients

Characteristics	Case No. (%)
Gender	
Male	196 (78.7)
Female	53 (21.3)
Age	
Range	31–82
Median	64

**Table 2:** Surgery characteristics of 249 patients

Characteristics	Case no. (%)
Type of gastric resection	
Total gastrectomy	115 (46.2)
Proximal gastrectomy	134 (53.8)
Surgical approach	
Left thoracotomy	73 (29.3)
Ivor Lewis	1 (0.4)
Transhiatal	175 (70.3)
Intraoperative blood transfusion	
Yes	231 (92.8)
No	18 (7.2)

**Table 3:** Pathology and postoperative characteristics of 249 patients

Characteristics	Case no. (%)
Type of pathology	
Adenocarcinoma	180 (72.3)
Adenocarcinoma with partial signet ring or mucinous	69 (27.7)
Tumor size	
> 4 cm	165 (66.3)
≤ 4 cm	84 (33.7)
N stage	
N0	71(28.5)
N1	42(16.9)
N2	49(19.7)
N3	87(34.9)
pTNM stage	
I	23 (9.2)
II	45 (18.1)
III	94 (37.8)
IV	87 (34.9)
Differentiation	
G1	2 (0.8)
G2	92 (36.9)
G3	155 (62.2)
Lymphovascular invasion	
Yes	127 (51)
No	122 (49)
Perineuronal invasion	
Yes	154 (61.8)
No	95 (38.2)
Adjuvant chemotherapy	
Yes	100 (40.2)
No	149 (59.8)
Number of metastatic lymph nodes	
Median (range)	3 (0-38)
PNLNR	
> 1	32 (12.9)
= 0	70 (28.1)
≤ 1	147 (59)
Postoperative hospital stays	
>10 d	133 (53.4)
≤ 10 d	116 (46.6)

*PNLNR* positive lymph nodes/ negative lymph nodes rate

Among the patients who underwent lymph node dissection, 70 (28.1%) patients had no positive lymph nodes, 32 (12.9%) patients had a PNLNR greater than 1, and 147 (59.0%) had a PNLNR less than 1. According to TNM staging, 23 (9.2%) patients were defined as stage I, 45 (18.1%) as stage II, 94 (37.8%) as stage III and 87 (34.9%) as stage IV.

In terms of surgery, there were a total of 3 types of surgical approaches, including 73 (29.3%) cases of left thoracotomy, 1 (0.4%) case of Ivor-Lewis surgery, and 175 (70.3%) cases of transhiatal surgery. Because only one patient underwent Ivor-Lewis surgery, we excluded this patient in the univariate and multivariate analyses. Total gastrectomy was performed in 115 (46.2%) patients, and proximal gastrectomy was performed in 134 (53.8%) patients. During surgery, a small portion of patients (n = 18) received intraoperative blood transfusions. After surgery, 53.4% patients stayed in the hospital for longer than 10 days, and 46.6% patients stayed for less than or equal to 10 days. Long hospital stays were mainly due to postoperative complications, postoperative nutritional status and several other reasons. Regarding adjuvant chemotherapy, 100 (40.2%) patients received adjuvant chemotherapy, and the main chemotherapy regimens were SOX (S-1 and oxaliplatin) and XELOX (capecitabine and oxaliplatin).

### Survival data and prognostic factors

**Fig. 1:**
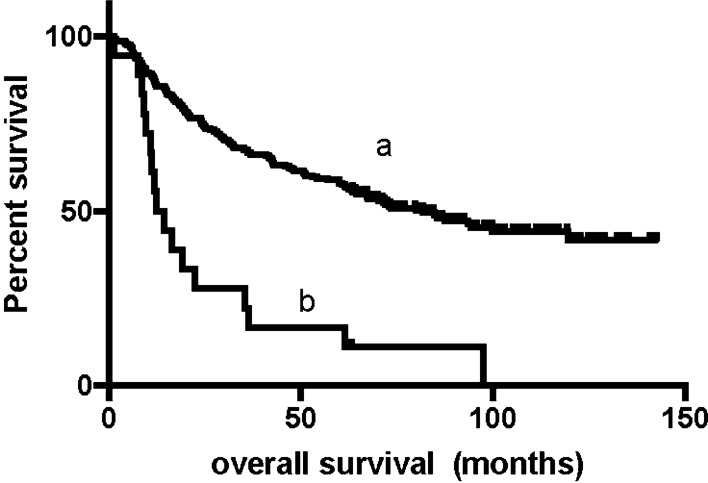
The mOS of patients without intraoperative blood transfusion is 81.8 m (**a**) and patients with intraoperative blood transfusion is 12.4 m (**b**), p < 0.001

**Fig. 2:**
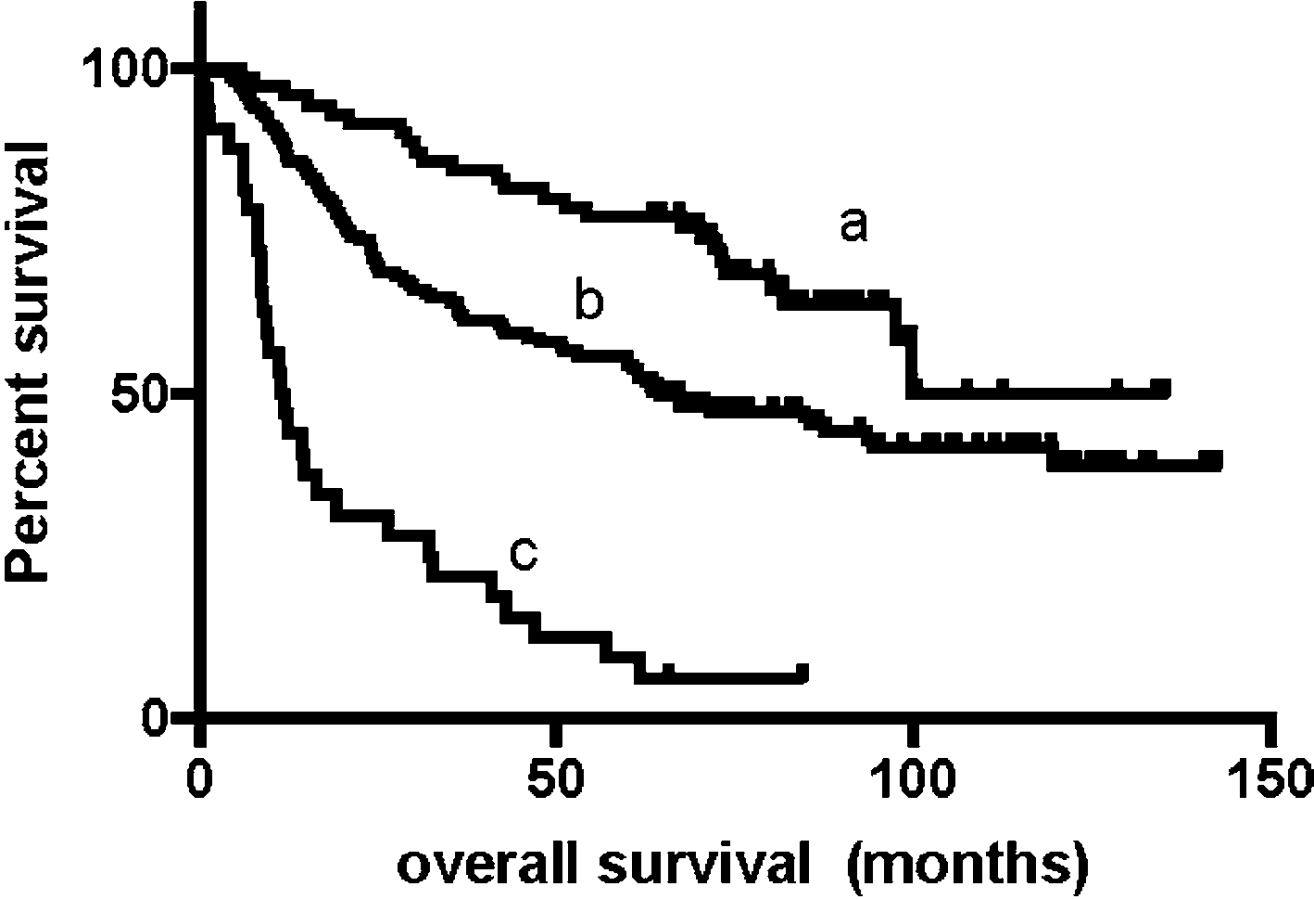
The mOS of patients with PNLNR > 1 was 1.9 m (**c**), paitents with PNLNR ≤ 1 (**b**) was 10.3 m, and patients with PNLNR = 0 had not reached (**a**), p < 0.001

**Fig. 3:**
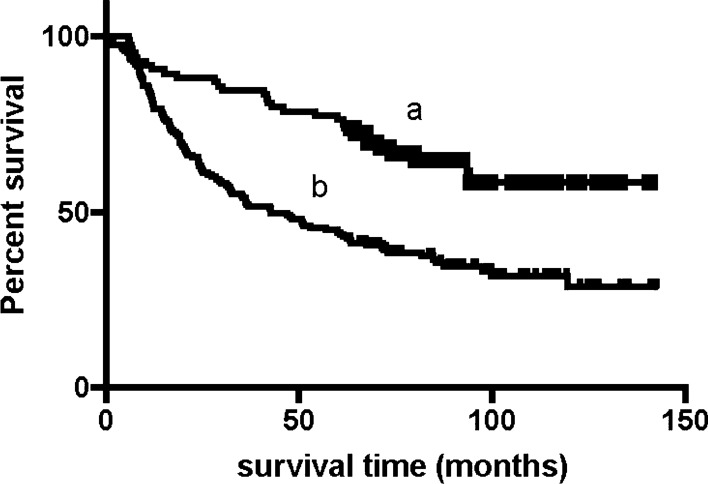
The median overall survival (mOS) of patients with tumor diameter more than 4 cm had not reached (**a**), and mOS of tumor diameter less or equal to 4 cm was 43.8 m (**b**), p < 0.001

We conducted our last follow-up in October 2019 by telephone interview or outpatient or inpatient department visits. The median follow-up time was approximately 75 months. At the last follow-up, 111 (44.6%) patients were still alive. The 1-year, 3-year and 5-year survival rates of all enrolled patients were 72%, 59%, and 49%, respectively. The median survival time (mOS) of these patients was 70.1 m (95% CI 53.6–86.6 m). Female patients had a significantly longer survival time than male patients (NA vs. 62.4 m, P = 0.039). Patients with pure adenocarcinoma had a significantly longer survival time than mixed pathology patients (85.4 vs. 42.5 m, P = 0.011). Patients with perineural invasion (NA vs. 48.1, P < 0.001) or lymphovascular invasion (NA vs. 40.9, P < 0.001) had shorter survival times, received fewer blood transfusions (12.4 vs. 81.8, P < 0.001) had a shorter survival time (Fig. 1). Patients who received adjuvant chemotherapy (61.7 vs. 93.4, P = 0.027) had a longer survival time. The median survival time of patients without lymph node metastasis was not reached, while the median survival time of N2 and N3 patients was 64.5 m and 24.0 m, respectively. Regarding the PNLNR, the mOS of patients with a PNLNR ≤ 1 was 67.2 m and that of patients with a PNLNR > 1 was 11.8 m (Fig. 2). The mOS of patients with a tumor size greater than 4 cm was also not reached, while the mOS of those with a tumor size less than or equal to 4 cm was 43.8 m (Fig. 3). pTNM stage was also significantly related to survival time. Patients with elevated CA199, CEA, CA242 and AFP levels had shorter survival times (Table 4). Only 1 of 249 patients died within 30 days after surgery.

**Table 4:** Univariate analysis of survival after surgery

Variables	MST (month)	Univariate analysis		P-value
		HR	95% CI	
Gender (male vs. female)	62.4 vs. NA	1.608	1.019–2.539	0.039
Pathology (adeno vs. mix)	85.4 vs. 42.5	0.632	0.442–0.903	0.011
Blood transfusion (no vs. yes)	12.4 vs. 81.8	0.262	0.156–0.440	< 0.001
Surgical approaches (left thoracotomy vs. transhiatal)	42.5 vs. 87.6	1.569	1.110–2.218	< 0.001
Tumor size (≤ 4cm vs. >4cm)	NA vs. 43.8	0.41	0.274–0.616	< 0.001
Adjuvant chemotherapy (no vs. yes)	61.7 vs. 93.4	1.477	1.042–2.094	0.027
Postoperative hospital stays (≤ 10d vs. > 10d)	59.9 vs. 99.7	0.806	0.680-0.956	0.012
Type of gastric resection (total vs. proximal)	73.5 vs. 64.5	0.96	0.811–1.135	0.63
PNLNR				
= 0	NA	0.403	0.298–0.543	
≤ 1	67.2	0.772	0.613–0.972	
> 1	11.8	1	1	
Perineuronal invasion (no vs. yes)	NA vs. 48.1	0.454	0.310–0.664	< 0.001
Lymphovascular invasion (no vs. yes)	NA vs. 40.9	0.448	0.317–0.634	< 0.001
Differentiation	80 vs. 73 vs. 63			0.851
pTNM stage				< 0.001
I	97.4	0.253	0.121–0.526	
II	99.7	0.265	0.155–0.451	
III	119.56	0.364	0.248–0.533	
IV	24.5	1	1	
Serum CA199 (U/ml) (< 37 vs. ≥ 37)	93.4 vs. 31.7	0.784	0.654–0.939	0.008
Serum CEA (ng/ml) (< 5 vs. ≥5)	80.0 vs. 26.3	0.74	0.623–0.878	< 0.001
Serum CA242 (U/ml) (< 20 vs. ≥20)	73.0 vs. 12.0	0.746	0.596–0.934	0.01
Serum CA125 (U/ml) (< 35 vs. ≥35)	71.4 vs. 70.1	0.605	0.423–0.866	0.004
Serum AFP (ng/ml) (< 10 vs. ≥10)	80.3 vs. 51.1	1.067	0.761–1.496	0.706
Serum CA724 (U/ml) (< 6.9 vs. ≥6.9)	70.1 vs. 80.0	0.862	0.715–1.039	0.117
Serum ferritin (ng/ml) (< 274.66 vs. ≥274.66)		0.952	0.708–1.280	0.745

The prognostic factors included age, sex, pathological type, tumor size, type of surgical approach, serum tumor biomarkers, and blood transfusion. Both univariate and multivariate analyses were applied (Tables 4 and 5). Among the factors related to survival in univariate analyses, 16 factors had significance, including sex, pathological type, intraoperative blood transfusion, surgical approach, and several serum tumor biomarkers. Factors including type of gastric resection, tumor differentiation, elevated serum AFP and serum ferritin had no significance. Then, multivariate analysis was conducted to identify the predictive indicators for a good prognosis using the parameters with a P value less than 0.5 in univariate analysis. Multivariate analysis showed that intraoperative blood transfusion, tumor size larger than 4 cm, no adjuvant chemotherapy, higher positive/negative lymph node ratio, perineural invasion, elevated serum CEA before surgery, and length of stay in the hospital greater than 10 days after surgery were independent risk factors for survival in resected Siewert type II AEG patients.

**Table 5:** Multiple cox regression analysis of survival after surgery

Variables	Multivariate analysis		P-value
	HR	95%CI	
Gender (male vs. female)	1.25	0.981–1.593	0.071
Pathology (adeno vs. mix)	1.066	0.864–1.314	0.55
Blood transfusion (no vs. yes)	0.63	0.471–0.843	0.002
Surgical approaches (left thoracotomy vs. transhiatal)	1.365	0.935–1.995	0.107
Tumor size (≤ 4cm vs. > 4cm)	0.728	0.582–0.912	0.006
Postoperative hospital stays (≤ 10d vs. > 10d)	0.833	0.694–0.999	0.048
PNLNR (> 1)	1	1	< 0.001
PNLNR (= 0)	0.561	0.268–1.175	0.126
PNLNR (≤ 1)	0.773	0.507–1.180	0.233
Perineuronal invasion (no vs. yes)	0.727	0.574–0.922	0.008
Lymphovascular invasion (no vs. yes)	0.899	0.721–1.121	0.346
pTNM stage IV	1	1	0.171
Stage III	1.166	0.541–2.512	0.694
Stage II	0.868	0.442–1.707	0.682
Stage I	0.764	0.426–1.369	0.366
Serum CA199 (U/ml) (<37 vs. ≥37)	0.986	0.784–1.239	0.902
Serum CEA (ng/ml) (<5 vs. ≥5)	0.768	0.623–0.947	0.014
Serum CA242 (U/ml) (<20 vs. ≥20)	0.934	0.707–1.233	0.629
Serum CA125 (U/ml) (<35 vs. ≥35)	0.739	0.490–1.114	0.148
Adjuvant chemotherapy (no vs. yes)	1.478	1.214–1.800	< 0.001

## Discussion

A number of various surgical approaches are available for Siewert type II AEG patients. With the increasing use of laparoscopic and thoracoscopic surgery, the surgical options have become more diversified. Various surgical approaches may lead to different numbers of dissected lymph nodes, and the surgical approach is strongly associated with prognosis.

Previous studies have suggested that the incidence of respiratory-related complications was noted to be higher in the thoracotomy group than in the transhiatal group [13–15]. In this study, only 1 patient underwent Ivor-Lewis surgery. There was no significant difference in postoperative length of hospital stay between the left thoracotomy group and the transhiatal group, with a median postoperative length of hospital stay of 11 days for both groups. However, survival was longer in the transhiatal group than in the thoracotomy group, which was consistent with previous similar studies [13]. Although no convincing theory so far can explain the difference between left thoracotomy and transhiatal thoracotomy, left thoracotomy is considered to be more invasive and has fewer survival benefits. This may be due to the much higher morbidity rate after thoracotomy, and an increasing number of studies have concluded that the transhiatal approach would be better for Siewert type 2 AEG patients.

In previous retrospective studies, perioperative blood transfusions were associated with poor prognosis after surgery for cancer and were a major independent risk factor for postoperative bacterial infection [16, 17]. Patients receiving intraoperative blood transfusion often have anemia before surgery or major intraoperative blood loss during surgery. This study found that the prognosis of patients who received intraoperative blood transfusions was poor. In this respect, laparoscopic surgery may be more advantageous as a minimally invasive surgical procedure.

When the number of positive lymph node metastases was included in the univariate analysis, it was concluded that patients with different lymph node stages had significant survival differences, but in the multivariate analysis, the number of lymph node metastases was not an independent risk factor for this conclusion. However, the ratio of positive lymph nodes to negative lymph nodes (PNLNR) was significant in both univariate and multivariate analyses. This may be because the number of lymph nodes dissected in different surgeries varies greatly, and mere comparisons of the number of positive lymph nodes cannot accurately assess the prognosis of patients; thus, PNLNR is a better predictor of prognosis.

Tumor size is a key factor affecting prognosis, and previous articles used a tumor diameter of 4 cm as the cutoff to analyze prognosis [18]. In this study, it was found that tumor size affected prognosis more than T stage and pTNM stage, and patients with a tumor size larger than 4 cm also had a worse prognosis. Moreover, adjuvant chemotherapy is quite important for AEG patients who undergo resection. Oxaliplatin-based systemic chemotherapy has led to significant survival benefits. Elevated serum CEA and perineural invasion in pathology were also prognostic factors, which reminds us to conduct thorough preoperative evaluations, including for serum tumor biomarkers, and create detailed pathological reports.

As our study is a single-center retrospective study, we could not avoid some biases from incomplete patient data and heterogenous surgical operations. Because the information on the time of recurrence was incomplete, we did not conduct statistical analysis on the median time of recurrence and did not analyze the factors affecting recurrence. Our study was unable to record all complications in detail; therefore, we can only indirectly reflect on the influence of patient recovery on prognosis based on the length of hospital stay. A larger study, preferably a randomized controlled trial in multiple centers, is needed to standardize the surgical options for Siewert type II AEG patients.

## Conclusions

Adjuvant chemotherapy, PNLNR, intraoperative blood transfusion, tumor size, perineural invasion, serum CEA, and duration of hospital stay after surgery are independent risk factors for survival in Siewert type II AEG patients who undergo resection. PNLNR is a better prognostic factor than pure N stage.

## Data Availability

The datasets used and/or analysed during the current study are available from the corresponding author on reasonable request.

## Abbreviations

AEG: Adenocarcinoma of the esophagogastric junction
PNLNR: Ratio of positive to negative lymph nodes
OS: Overall survival
